# Maternal Arsenic Exposure and Impaired Glucose Tolerance during Pregnancy

**DOI:** 10.1289/ehp0800533

**Published:** 2009-03-11

**Authors:** Adrienne S. Ettinger, Ami R. Zota, Chitra J. Amarasiriwardena, Marianne R. Hopkins, Joel Schwartz, Howard Hu, Robert O. Wright

**Affiliations:** 1 Department of Environmental Health, Harvard School of Public Health, Boston, Massachusetts, USA; 2 Channing Laboratory, Department of Medicine, Brigham and Women’s Hospital, Harvard Medical School, Boston, Massachusetts, USA; 3 University of Michigan School of Public Health, Ann Arbor, Michigan, USA; 4 Department of Medicine, Children’s Hospital Boston, Boston, Massachusetts, USA

**Keywords:** arsenic, gestational diabetes, impaired glucose tolerance, pregnancy, Superfund

## Abstract

**Background:**

Accumulating evidence has shown an increased risk of type 2 diabetes in general populations exposed to arsenic, but little is known about exposures during pregnancy and the association with gestational diabetes (GD).

**Objectives:**

We studied 532 women living proximate to the Tar Creek Superfund Site to investigate whether arsenic exposure is associated with impaired glucose tolerance during pregnancy.

**Methods:**

Blood glucose was measured between 24 and 28 weeks gestation after a 1-hr oral glucose tolerance test (GTT) as part of routine prenatal care. Blood and hair were collected at delivery and analyzed for arsenic using inductively coupled plasma mass spectrometry with dynamic reaction cell.

**Results:**

Arsenic concentrations ranged from 0.2 to 24.1 μg/L (ppb) (mean ± SD, 1.7 *±*1.5) and 1.1 to 724.4 ng/g (ppb) (mean ± SD, 27.4 *±* 61.6) in blood and hair, respectively. One-hour glucose levels ranged from 40 to 284 mg/dL (mean ± SD, 108.7 *±* 29.5); impaired glucose tolerance was observed in 11.9% of women when using standard screening criterion (> 140 mg/dL). Adjusting for age, Native-American race, prepregnancy body mass index, Medicaid use, and marital status, women in the highest quartile of blood arsenic exposure had 2.8 higher odds of impaired GTT than women in the lowest quartile of exposure (95% confidence interval, 1.1–6.9) (*p*-trend = 0.008).

**Conclusions:**

Among this population of pregnant women, arsenic exposure was associated with increased risk of impaired GTT at 24–28 weeks gestation and therefore may be associated with increased risk of GD.

Arsenic exposure is a well-recognized public health problem: Millions of people worldwide are potentially exposed predominantly to inorganic arsenic from drinking water contaminated by naturally occurring sources (Tchounwou et al. 1999). Chronic exposure to arsenic is associated with a number of adverse health effects (Yoshida et al. 2004).

Accumulating evidence from epidemiologic and experimental studies has shown an increased risk of type 2 diabetes in populations with high exposure to arsenic (Longnecker and Daniels 2001; Navas-Acien et al. 2006; Tseng et al. 2002). One recent study found an association between arsenic exposure and diabetes in a nationally representative sample of U.S. adults (Navas-Acien et al. 2008). Arsenic-induced diabetes may occur through induction of insulin resistance and beta-cell dysfunction by arsenic (or its methylated metabolites) via induction of oxidative stress or interferences in signal transduction or gene expression (Tseng 2004). Individual factors (e.g., nutritional status, genes) may also influence arsenic toxicity (Vahter 2007).

Few studies have explored the effects of arsenic on human pregnancy outcomes (Huyck et al. 2007; Rahman et al. 2007; Vahter et al. 2006), and none have investigated risk of diabetes in pregnant women, even though diabetes is a major potential complication of pregnancy with adverse effects for both mothers and infants. Gestational diabetes (GD) occurs when resistance to circulating insulin leads to hyperglycemia, and this impaired glucose metabolism is first detected during pregnancy. GD has an estimated prevalence of 1–14% of all pregnancies depending on race/ethnicity and diagnostic criteria used [American Diabetes Association (ADA) 2004; Ferrara 2007]. Although there is some controversy over the definitive screening criteria, GD is usually first identified by testing a women’s blood glucose level 1 hr after receiving a 50-g oral glucose challenge between 24 and 28 weeks gestation as part of routine prenatal care [American College of Obstetrics and Gynecology (ACOG) 2001].

GD is associated with 30–60% increased risk of developing diabetes in later life in the mother and also poses intergenerational risks to the fetus (Metzger 2007). Diabetes in pregnancy is associated with increased risk of major congenital malformations, macrosomia (birth weight > 4,000 g or > 90th percentile for gestational age), and complications during delivery and in the perinatal period including stillbirth (Fetita et al. 2006). Infants born to mothers with impaired glucose tolerance/GD are at increased risk of subsequent impaired glucose tolerance and obesity. However, there is evidence that early intervention and treatment, such as dietary counseling, blood glucose monitoring, and insulin or other drugs (if appropriate), may lead to improved outcomes for mother and child (World Health Organization 2006). Therefore, it is an important public heath priority to identify risk factors for GD.

We examined maternal arsenic exposure and risk of impaired glucose tolerance during pregnancy, which is an important determinant of GD, in a population of pregnant women living in an area surrounding the Tar Creek Superfund site in Ottawa County, Oklahoma.

## Materials and Methods

### Study location

The Tar Creek Superfund site, a former lead and zinc mining area, occupies 40 square miles of land in northeastern Oklahoma that is heavily contaminated with metals from mining waste [Agency for Toxic Substances and Disease Registry (ATSDR) 2004]. It is part of the former Tri-State Mining District, which extended from Oklahoma through southeast Kansas and into southwest Missouri. In 1983, under the Comprehensive Environmental Response, Compensation, and Liability Act (“Superfund”) legislation (1980), the site was added to the National Priority List because of extensive metal contamination of surface water, groundwater, sediments, and soils (ATSDR 2004). Approximately 30,000 people live in the area surrounding the site, including many residents of Native-American descent on allotted Indian land. Massive piles of mine tailings, known locally as “chat,” contain elevated concentrations of lead, zinc, and other metals (Schaider et al. 2007). Many of the original chat piles have been partially excavated and sold as fill for construction projects including roadways, playgrounds, residential driveways, and foundations of private homes (ATSDR 2004). In addition, at least 25% of drinking water samples in northeastern Oklahoma contain > 10 μg/L arsenic (Ryker 2001; U.S. Geological Survey 2007).

### Study subjects

Subjects were participants in an ongoing prospective birth cohort study of biological markers of fetal and early childhood exposure to metals mixtures, maternal psychosocial stress, and their impact on neurocognitive development. This research is being conducted in the area of the Tar Creek Superfund site as a collaborative effort between the Harvard School of Public Health (HSPH), the Local Environmental Action Demanded (L.E.A.D.) Agency (a community-based nonprofit organization), and the Integris Baptist Regional Health Center, as part of the Center for Children’s Environmental Health and Disease Prevention Research at HSPH. The research protocol was approved by the Human Subjects Committees of Integris Health and Harvard School of Public Health and complied with all federal regulations governing protection of human subjects. All participants received a detailed explanation of the study before signing an approved written informed consent form. Interviewer-administered questionnaires and medical records review were used to obtain information about sociodemographic characteristics, potential environmental, occupational, and recreational sources of exposures, psychosocial stress, and details about the index pregnancy.

Pregnant women were recruited during pre-natal visits or upon admission for labor and delivery at the Integris Baptist Regional Health Center in Miami, Oklahoma, which is the only hospital in Ottawa County. To be eligible for participation in the cohort study, women must have met the following criteria: *a*) giving birth to a live-born infant at Integris hospital; *b*) intention to live within the study area (Ottawa County) for the next 2 years; *c*) not currently enrolled in the study with another child; and *d*) English language proficiency that would allow ability to read, understand, and participate in informed consent process in English. There were 2,312 births recorded at the hospital from 1 November 2002 to 15 August 2008, and a total of 589 women were enrolled in the cohort during that same period. Because the birth cohort study was a continuation of a smaller research endeavor, detailed data on enrollment and eligibility are not available for the entire study period. However, the exclusion criteria were designed to be as inclusive as possible, and the most common reasons for ineligibility were that women were already enrolled with another child or did not live (or were not intending to stay) in the area. Participants who were missing information on blood arsenic (*n* = 23) or blood glucose (*n* = 34) were excluded, leaving a total of 532 women included in this analysis. In a subset of these women with hair samples available (*n* = 179; 34%), we conducted analyses using hair arsenic as a biomarker of exposure. The main reason for lack of an appropriate hair sample was that the participant had chemically treated hair.

### Laboratory measurements

Maternal blood and hair samples were collected at delivery (± 12 hr) and analyzed for total arsenic concentration at the HSPH Trace Metals Laboratory (Boston, MA).

Hair was collected from the back of the head using high-grade stainless steel scissors to cut as close to the scalp as possible from all participants who had chemically untreated (“virgin”) hair (*n* = 179; 34%), which was requested to be free of any gels, oils, creams, sprays, or other styling products. Collection was done within a clean environment to avoid the introduction of external contaminants before, during, and after the collection process, and the following guidelines were maintained to ensure the collection of a metabolically representative sample. Approximately 2 inches of virgin hair (representing the previous 5 months of exposure) was collected and placed directly into a small manila coin envelope provided by the laboratory and then sealed with the glue flap only. The weight required for the hair sample analysis is 0.125 g, which corresponds to about 50 strands of hair or the thickness of a pencil. Total hair arsenic concentration was measured using previously published methods (Chen et al. 1999). The limit of detection for hair arsenic was 0.2 ng/g.

Whole blood was collected in trace element–free tubes [BD Vacutainer royal blue top, venous blood collection tubes with K2EDTA #368381 (Becton Dickinson, Franklin Lakes, NJ)]. One milliliter of blood was transferred into a 15-mL plastic tube, digested with 2 mL concentrated HNO_3_ acid (Optima, Seastar Chemical Co., Pittsburgh, PA) for 24 hr, and then diluted to 10 mL with deionized water after adding 1 mL 30% hydrogen peroxide (Ultrex Ultrapure Reagents, J.T. Baker, Phillipsburg, NJ). Samples were further diluted as needed. Acid-digested samples were then analyzed by inductively coupled plasma mass spectrometry with dynamic reaction cell (Elan 6100; PerkinElmer, Norwalk, CT), using oxygen as the reaction gas. Concentration of arsenic was estimated using external calibration with indium as the internal standard. The limit of detection for blood arsenic was 0.2 μg/L.

Quality control measures for both blood and hair included analysis of the following: an initial calibration verification standard [National Institute of Standard and Technology (NIST; Gaithersburg, MD) standard reference material #1643e (trace elements in water)]; a solution of 1 ng/mL standard solution of arsenic (NIST traceable); continuous calibration standards; procedural blanks; and certified reference material GBW-07601 (human hair; Institute of Geophysical and Geochemical Exploration, Langfang, China). Results were reported as the average of five replicate measurements per sample, and samples with relative standard deviation > 30% for the five replicates were flagged. Recovery of the analysis of quality control standard by this procedure is 90–110%, and coefficient of variation of the within-day analysis was ~ 0.05. The between-assay coefficient of variation for arsenic was 0.1. Samples with blood arsenic concentration < 0.2 μg/L (*n* = 4; 0.7%), hair arsenic concentration < 0.2 ng/g (*n* = 4; 2%), or insufficient sample weight for analysis (*n* = 18; 10%) were reported as less than the limit of detection and not included in the analysis.

Maternal blood (plasma) glucose was measured at a prenatal visit between 24 and 28 weeks gestation after a 1-hr, 50-g oral glucose tolerance test (GTT) as part of routine prenatal care (ACOG 2001), and results were obtained from the medical record. All GTTs required that the patient be fasting after midnight the day before the administration of the test. Glucose was measured in plasma using the Beckman Coulter CX 9 (Beckman Coulter, Inc. Fullerton, CA) at the Integris Baptist clinical laboratory.

### Statistical analysis

Univariate and bivariate summary statistics and distributional plots were examined for all variables. Spearman’s correlation between arsenic variables and blood glucose were examined. The arsenic exposure measurements were grouped into quartiles and entered into the models as dummy variables, with the lowest quartile used as the reference category. Impaired glucose tolerance after the 1-hr, 50-g oral GTT was defined using standard screening criteria as a blood glucose level of > 140 mg/dL (7.8 mmol/L), which provides sensitivity to identify approximately 80% of women with GD, and also using a more sensitive value of > 130 mg/dL (7.2 mmol/L), which identifies approximately 90% of women with GD (ADA 2004).

We used logistic regression to model prediction of the probability of occurrence of impaired glucose tolerance in relation to arsenic exposure including the major risk factors for GD [age, race/ethnicity, prepregnancy body mass index (BMI)] as the main covariates of interest. Covariates of interest were determined *a priori* based on biological considerations, and other covariates (marital and insurance status) were added using statistical considerations if they were significant (*p* = 0.1) in bivariate models. Maternal age (centered) and centered age-squared were included in the models, because maternal age showed a U-shaped association in exploratory smooth plots. The linear test for trend [degrees of freedom (df) = 1] across exposure categories was conducted by assigning the quartiles of exposure values of 0, 1, 2, 3 (ordinal variable) and specifying an appropriate linear combination vector (with a coefficient sum that equals 0) in a contrast statement. Linear models were also fitted using arsenic exposure as a continuous variable and presented for an IQR increase in exposure. To account for exposure measurement error and thus decrease any downward bias in the effect estimates, models were weighted by the inverse of measurement error (exposure) variance of the five laboratory replicates per sample. In a subset analysis, women reporting a previous history of diabetes (*n* = 20) or taking medications for diabetes (*n* = 2)—together labeled “history of diabetes”—were excluded from the analysis. To explore potential nonlinear associations between arsenic and blood glucose levels, we used generalized additive models with penalized splines (df = 3) for the arsenic exposure variable weighted by the inverse of the variance of the exposure variable. All analyses were conducted using Statistical Analysis System (SAS version 9.1; SAS Institute Inc., Cary, NC) and R, version 2.2.0 (The R Foundation for Statistical Computing, Boston, MA).

## Results

Maternal characteristics and their bivariate associations with impaired glucose tolerance are presented in Table 1. The mean (± SD) age of women in this cohort was 24.5 ± 5.4 years with 7% ≤ 18 years of age and almost 5% > 35 years of age. Approximately 74% graduated from high school, and most of the study cohort (81%) received public health insurance through SoonerCare, the Oklahoma State Medicaid program, which has inclusive eligibility guidelines for pregnant women and children. More than one-third (37%) reported smoking cigarettes at some time during their pregnancy, and more than half the women (59%) were considered overweight or obese prior to pregnancy. Although most of the population was Caucasian (66%), women of Native American ancestry comprised almost 24% of the cohort.

Blood arsenic concentration ranged from 0.2 to 24.1 μg/L [parts per billion (ppb)] (*n* = 532; mean 1.7 ± 1.5) and was significantly correlated with blood glucose level (Spearman’s rho = 0.1; *p* = 0.02). Hair arsenic concentration, available on a subset of participants, ranged from 1.1 to 724.4 ng/g (ppb) (*n* = 180; mean 27.4 ± 61.6) and was not significantly correlated with blood glucose level (Spearman’s rho = 0.04; *p* = 0.6). Among the subset of women with both exposure bio-markers available (*n =* 179), blood and hair arsenic levels were not significantly correlated with each other (Spearman’s rho = −0.13; *p* = 0.08). One-hour glucose levels ranged from 40 to 284 mg/dL (mean, 108.7 ± 29.5). Almost 12% of women participating had impaired GTTs (blood glucose > 140 mg/dL) at the 24- to 28-week prenatal visit. Using a more sensitive screening criterion (blood glucose > 130 mg/dL), > 20% had impaired GTT results.

In bivariate analyses, maternal characteristics significantly associated with higher blood arsenic levels were increasing maternal age, not having graduated from high school, use of public health assistance, married or living with partner, Native-American race/ethnicity, multiparity, higher prepregnancy weight and BMI, self-reported history of diabetes or high blood pressure, and having a cesarean-section delivery, whereas lower blood arsenic concentration was associated with taking prenatal vitamins (data not shown).

In multivariate analyses (*n* = 456), adjusting for age, Native-American race/ethnicity, prepregnancy BMI, Medicaid use, and marital status, women in the highest quartile of blood arsenic exposure had 2.79 higher odds of impaired GTT (> 140 mg/dL) than women in the lowest quartile of exposure [95% confidence interval (CI), 1.13–6.87] (Table 2). There was a statistically significant trend in risk of impaired GTT by increasing quartile of exposure (*p*-trend = 0.008). Using the more sensitive criterion (> 130 mg/dL), women in the highest quartile of blood arsenic exposure had 2.35 higher odds of impaired GTT (> 130 mg/dL) than women in the lowest quartile of exposure (95% CI, 1.18–4.69) (*p*-trend = 0.006).

In models using arsenic exposure as a continuous variable and accounting for exposure measurement error (weighted by the inverse of exposure variance), an IQR increase in blood arsenic concentration was associated with 1.65 times higher odds of impaired GTT (> 140 mg/dL) (95% CI, 1.52–1.79) and with 1.73 higher odds of impaired GTT (> 130 mg/dL) (95% CI, 1.61–1.87) (Table 2). Before the measurement error correction technique, an IQR increase in blood arsenic concentration was associated with 1.52 times higher odds of impaired GTT (> 140 mg/dL) (95% CI, 1.18–1.97) and with 1.42 higher odds of impaired GTT (> 130 mg/dL) (95% CI, 1.13–1.77) (data not shown).

Self-reported history of diabetes (*n* = 20) or taking medications for diabetes (*n* = 2) (“history of diabetes”) was a strong predictor of impaired glucose tolerance at the 24- to 28-week prenatal visit in the bivariate analysis [odds ratio (OR) = 4.70; 95% CI, 1.89–11.70] (Table 1). However, because we did not have physician-confirmed diagnoses and were relying on self-report, and because the occurrence of preexisting diabetes may also be causally related to arsenic exposure (and thus an intervening variable), we chose not to include this variable in the main models. When we restricted the models to include only women without self-reported history of diabetes, not taking medications for diabetes, and with other covariates available (*n* = 439), the effect of arsenic exposure remained, as did the statistically significant trend. An interquartile range (IQR) increase in exposure (1.2 μg/L) was associated with 1.76 higher odds of impaired GTT (> 140 mg/dL) (95% CI, 1.61–1.93) (*p*-trend = 0.04) and 1.83 higher odds of impaired GTT (> 130 mg/dL) (95% CI, 1.69–1.98) (*p*-trend = 0.02).

We repeated these models in the subset of women with hair arsenic concentrations and the covariates of interest available (*n* = 149). Women in the highest quartile of hair arsenic exposure had 4.20 higher odds of impaired GTT (> 140 mg/dL) (95% CI*,* 0.74–23.86) than women in the lowest quartile of exposure, although these results were not statistically significant (Table 3). Among subjects with no history of diabetes (*n* = 143), an IQR increase in exposure (15.3 ng/g) was associated with 2.70 higher odds of impaired GTT (> 140 mg/dL) (95% CI*,* 0.59–12.42) (*p*-trend = 0.11) and 1.55 higher odds of impaired GTT (> 130 mg/dL) (95% CI, 0.44–5.44) (*p*-trend = 0.41).

**Figure 1:**
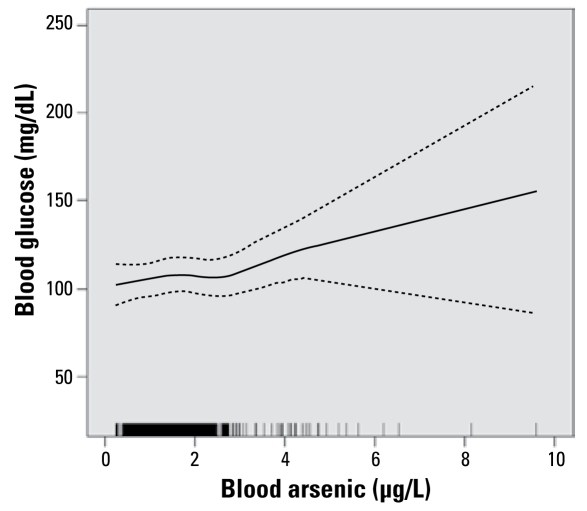
Adjusted dose–response relationship for the effect of blood arsenic (μg/L) on blood glucose (mg/dL) from a generalized additive model with penalized splines (df = 3, *p* = 0.01) adjusted for age (centered), centered age-squared, prepregnancy BMI, Native-American race/ethnicity, use of Medicaid insurance, and married or living with partner; weighted by the inverse of the variance of exposure variable (*n* = 455). Dashed lines represent 95% CIs. Vertical bars along the abscissa mark the observed values of blood arsenic for individual subjects.

Figure 1 shows the adjusted dose–response relationship for the effect of blood arsenic (micrograms per liter) on blood glucose (milligrams per deciliter) from a generalized additive model.

## Discussion

Among this population of pregnant women with relatively low exposures, arsenic concentration was associated with impaired glucose tolerance during pregnancy and therefore may be associated with increased risk of GD. Women in the highest quartile of blood arsenic exposure had almost three times higher odds of impaired GTT than women in the lowest quartile of exposure, and there was a statistically significant trend in risk of impaired GTT by increasing quartile of exposure. The prevalence of impaired glucose tolerance during pregnancy in this population was high (12–20%, depending on criteria used) and may be related to high prevalence of several known risk factors for GD observed in this population. When we restricted the analyses to women with no self-reported history of diabetes, the association of arsenic exposure on impaired glucose tolerance remained. The effect of the measurement error correction technique applied was, as expected, to reduce the downward bias in the effect estimates due to nondifferential misclassification of exposure by the laboratory.

Environmental exposures may result in abnormal glucose metabolism, which in turn increases the risk of developing diabetes, through several plausible mechanisms (Longnecker and Daniels 2001). Arsenic is a known endocrine disruptor (Tseng 2004) and may disrupt the glucocorticoid receptor (Kaltreider et al. 2001), which regulates a wide range of biological processes in humans, including insulin sensitivity. It is also possible that some aspect of having diabetes or prediabetes alters arsenic metabolism in such as way as to cause higher levels in the body. However, experimental evidence suggests that oxidative stress and insulin resistance can be induced by arsenic, suggesting a biological plausibility to arsenic-induced diabetes (Tseng et al. 2004).

In a recent analyses of NHANES (National Health and Nutrition Examination Survey) data, average urinary total arsenic concentrations were 8.30 μg/L (95% CI, 7.19–9.57) (Caldwell et al. 2008). Using data from the same nationally representative cross-sectional survey of the U.S. population, Navas-Acien and colleagues (2008) found that participants with type 2 diabetes had a 26% higher level of total arsenic (95% CI, 2.0–56.0) and the OR for type 2 diabetes comparing participants at the 80th versus the 20th percentiles was 3.58 for the level of urinary total arsenic (95% CI, 1.18–10.83). One previous study has shown an association with environmental exposures (agricultural pesticides) and increased risk of GD among wives of licensed pesticide applicators (Saldana et al. 2007); however, that study relied on self-report of GD and pesticide usage.

In this study, we used sensitive laboratory biomarkers of both the exposure (arsenic) and outcome (plasma glucose). Levels of total arsenic exposure (measured in maternal blood and hair) in our study were higher than reported background levels of arsenic in unexposed individuals (ATSDR 2007). Exposure at hazardous waste sites may occur by a variety of pathways, including inhalation of dusts in air, ingestion of contaminated soil or water, or through the food chain. We do not have data on speciated arsenic available to differentiate between inorganic and organic arsenic exposure. However, these results taken together suggest that if our findings are confirmed, a significant portion of U.S. women of childbearing age may be at risk for GD due to environmental exposures such as arsenic.

One limitation of our study was that we used blood collected at the time of delivery as a biomarker of arsenic exposure, which was measured after the outcome measurement (blood glucose). Because arsenic binds to the sulfhydryl groups in keratin, the primary component in hair and nails, this is where the highest levels of arsenic tend to be found (Liu et al. 2008). Therefore, studies of chronic arsenic exposure often use hair or nails as biomarkers of cumulative exposure. Although nails or hair may best represent cumulative exposure, keratin-bound arsenic is isolated from the body’s further metabolic processes and may be less biologically active than blood arsenic. Because of these differences in biomarkers of arsenic exposure, we used hair, in a subset of women with samples available, to provide estimates of longer-term exposures, with every 1 cm of hair representing each month of prior exposure (Kurttio et al. 1998). We found similar results, though not statistically significant, to those from our analyses using blood arsenic. We believe this is because with chronic exposures, blood arsenic likely reaches a steady-state concentration and therefore may better reflect an individual’s total internal arsenic dose (Hall et al. 2006).

In our study, in the subset of women with both samples available, blood and hair arsenic concentrations were not correlated. However, blood arsenic concentration has previously been found to correlate both with other biological matrices and with environmental sources of arsenic. In a Bengali population with much higher levels of exposure, blood arsenic levels were highly correlated with creatinine-adjusted urinary concentrations (*r* = 0.85) and with drinking-water arsenic concentration (*r* = 0.75) (Hall et al. 2006). In addition, blood arsenic also correlated with arsenic metabolites including methylated forms, which may represent the most toxic arsenic species (Hall et al. 2007).

Another limitation of our study is that we used the results of GTTs performed as part of routine prenatal care and not conducted as part of a structured research protocol. We used blood glucose measured after a fasting, 50-g oral glucose challenge test, which is the recommended standard of care (ACOG 2001), to identify women with impaired glucose tolerance who should go on to receive further testing to diagnose GD. We found similar results when using two threshold criteria for defining impaired glucose tolerance (> 140 and > 130 mg/dL). In addition, all of our tests were conducted and analyzed at one clinical laboratory facility and conducted independently of arsenic concentration, making any misclassification of the outcome likely nondifferential with respect to exposure and thus biasing our results toward the null hypothesis of no effect.

Our results likely represent more than a potential causal association between arsenic and GD but may also provide clues to long-observed health disparities that have been resistant to public health interventions. It is well recognized that there are many diseases and disorders that disproportionately affect the health of racial and ethnic minority populations in the United States. Women from minority groups, especially Native-American women, are much more likely to have type 2 diabetes during their childbearing years (National Institute of Diabetes and Digestive and Kidney Diseases 2006, 2008). In addition, these groups may be disproportionately affected by exposure to environmental contaminants due to socioeconomic conditions and cultural practices that use locally grown plants and animals and may differentially increase exposure when environmental contamination occurs (Harris and Harper 1997). We found Native-American race to be a predictor, although not statistically significant, of impaired glucose tolerance in pregnancy. Also, Ottawa County is an economically deprived area with a poverty rate higher than the Oklahoma state and national averages (Oklahoma County Health Care Authority 2005). Most of our cohort received public health insurance through SoonerCare, the Oklahoma State Medicaid medical benefits program, which has more lenient eligibility guidelines than federal standards, allowing more pregnant women and children to access health care. These findings highlight the need to identify and ameliorate racial, socioeconomic, and environmental disparities that may be adversely affecting the health of the particularly vulnerable populations such as pregnant women and children (Silbergeld and Patrick 2005).

In summary, GD is a major potential complication of pregnancy associated with negative health effects for both the mother and infant. Understanding the effects of environmental exposures on impaired glucose tolerance during pregnancy may have substantial public health importance beyond the direct effects on GD. Studies are needed to investigate environmental or behavioral factors that may contribute to risk for development and progression of diabetes, obesity, and its complications. Such studies should incorporate culturally specific lifestyle factors into treatment and prevention strategies to reduce risk across racial, ethnic, and socioeconomic groups. Future research in this area will be important for understanding whether different pathophysiologic mechanisms or risk factors are responsible for increased obesity and diabetes risk, especially in children. In addition, better understanding of modifiable risk factors for GD such as diet and activity patterns related to environmental exposures may lead to efforts at primary prevention.

## Figures and Tables

**Table 1:** Distribution of maternal characteristics and their univariate associations with impaired glucose tolerance^a^ at 28 weeks gestation, Ottawa County, Oklahoma, 2002–2008 (*n* = 532).

Characteristic	No. (%)^b^	OR (95% CI)
Age (years)		

14–18	39 (7.4)	0.54 (0.12–2.38)
19–24	275 (52.0)	1.00 (referent)
25–35	192 (36.2)	2.00 (1.14–3.50)
≥ 36	25 (4.7)	1.91 (0.61–5.99)

Education		

< 12th grade	140 (26.4)	1.35 (0.76–2.38)
≥ 12th grade	391 (73.6)	1.00 (referent)

Insurance status		

State Medicaid	427 (80.7)	1.46 (0.68–3.08)
Private	102 (19.3)	1.00 (referent)

Marital status		

Married or living with partner	326 (63.2)	2.41 (1.27–4.57)
Never married/separated/divorced	190 (36.8)	1.00 (referent)

Race/ethnicity		

White	340 (66.2)	1.00 (referent)
Native American	121 (23.5)	1.27 (0.70–2.32)
Other	53 (10.3)	0.29 (0.07–1.22)

Parity (no.)		

1	212 (39.96)	1.00 (referent)
2	170 (32.2)	1.42 (0.76–2.67)
≥ 3	148 (27.84)	1.34 (0.69–2.59)

Prepregnancy BMI (kg/m^2^)		

< 25	196 (41.4)	1.00 (referent)
25–30	145 (30.7)	1.61 (0.76–3.42)
≥ 30	132 (27.9)	3.50 (1.76–6.95)

Weight gain during pregnancy (kg)		

< 10	169 (35.7)	1.00 (referent)
10–15	133 (28.1)	0.83 (0.42–1.63)
>15	171 (36.2)	0.67 (0.34–1.29)

Self-reported history of diabetes		

Yes	22 (4.2)	4.70 (1.89–11.70)
No	507 (95.8)	1.00 (referent)

Self-reported history of high blood pressure		

Yes	34 (6.43)	2.03 (0.85–4.88)
No	495 (93.6)	1.00 (referent)

Smoked during pregnancy		

Yes	196 (37.0)	0.93 (0.53–1.61)
No	333 (63.0)	1.00 (referent)

Prenatal vitamin use		

Yes	354 (66.7)	1.00 (referent)
No	177 (33.3)	0.92 (0.52–1.62)

Blood arsenic (μg/L)		

Q1 (0.23–0.92)	133 (25)	1.00 (referent)
Q2 (0.93–1.39)	133 (25)	1.24 (0.50–3.10)
Q3 (1.40–2.08)	133 (25)	2.88 (1.28–6.49)
Q4 (2.09–24.07)	133 (25)	2.44 (1.07–5.58)

Hair arsenic (ng/g)		

Q1 (1.10–8.81)	44 (25)	1.00 (referent)
Q2 (8.93–13.11)	45 (25)	1.25 (0.31–5.00)
Q3 (13.26–24.12)	45 (25)	2.16 (0.60–7.78)
Q4 (24.22–724.41)	45 (25)	1.84 (0.50–6.80)

Q1–4, quartiles 1–4.

a Impaired glucose tolerance defined as 0: ≤ 140, 1: > 140 mg/dL.

b Data were missing for insurance status (*n* = 2), marital status (*n* = 15), race/ethnicity (*n* = 17), parity (*n* = 1), prepregnancy BMI (*n* = 58), weight gain during pregnancy (*n* = 58), self-reported history of diabetes (*n* = 2) or high blood pressure (*n* = 2), smoked during pregnancy (*n* = 2), hair arsenic (*n* = 353).

**Table 2:** Risk of impaired glucose tolerance at 28 weeks gestation using two threshold criteria, by blood arsenic exposure.^a^

	All subjects (*n* = 456)		Excluding subjects with history of diabetes (*n* = 439)	
Blood arsenic (μg/L)	OR^b^ (95% CI)	OR^c^ (95% CI)	OR^b^ (95% CI)	OR^c^ (95% CI)
Quartile				

Q1 (0.23–0.92)	1.00 (referent)	1.00 (referent)	1.00 (referent)	1.00 (referent)
Q2 (0.93–1.39)	1.02 (0.39–2.69)	1.03 (0.50–2.10)	1.07 (0.39–2.98)	1.00 (0.48–2.10)
Q3 (1.40–2.08)	2.65 (1.12–6.36)	2.21 (1.14–4.29)	2.83 (1.14–7.02)	2.23 (1.14–4.38)
Q4 (2.09–24.07)	2.79 (1.13–6.87)	2.35 (1.18–4.69)	2.46 (0.91–6.62)	2.08 (1.01–4.27)
*p*-trend	0.008	0.006	0.04	0.02

Continuous^d^				

IQR (1.2)	1.65 (1.52–1.79)	1.73 (1.61–1.87)	1.69 (1.54–1.84)	1.79 (1.65–1.93)

a Adjusted for maternal age (years), maternal age squared, prepregnancy BMI, Native-American race/ethnicity, Medicaid insurance, married or living with partner.

b Elevated glucose defined as 0: < 140, 1: > 140 mg/dL.

c Elevated glucose defined as 0: < 130, 1: > 130 mg/dL.

d Weighted by the inverse of variance of exposure variable; one extreme outlier removed.

**Table 3:** Risk of impaired glucose tolerance at 28 weeks gestation using two threshold criteria, by hair arsenic exposure.^a^

	All subjects (*n* = 149)		Excluding subjects with history of diabetes (*n* = 143)	
Hair arsenic (ng/g)	OR^b^ (95% CI)	OR^c^ (95% CI)	OR^b^ (95% CI)	OR^c^ (95% CI)
Quartile				

Q1 (1.10–8.81)	1.00 (referent)	1.00 (referent)	1.00 (referent)	1.00 (referent)
Q2 (8.93–13.11)	3.97 (0.62–25.37)	0.96 (0.27–3.38)	4.51 (0.36–56.55)	0.67 (0.17–2.75)
Q3 (13.26–24.12)	5.77 (0.98–33.88)	2.44 (0.78–7.63)	13.24 (1.27–138.62)	2.48 (0.75–8.19)
Q4 (24.22–724.41)	4.20 (0.74–23.86)	1.00 (0.31–3.29)	8.62 (0.87–85.20)	1.11 (0.32–3.78)
*p*-trend	0.40	0.81	0.11	0.41

Continuous^d^				

IQR (15.3)	2.32 (0.52–10.39)	1.50 (0.41–5.49)	2.70 (0.59–12.42)	1.55 (0.44–5.44)

a Adjusted for maternal age (years), maternal age squared, prepregnancy BMI, Native-American race/ethnicity, Medicaid insurance, married or living with partner.

b Elevated glucose defined as 0: ≤ 140, 1: > 140 mg/dL.

c Elevated glucose defined as 0: ≤ 130, 1: > 130 mg/dL.

d Weighted by the inverse of variance of exposure variable; one extreme outlier removed.
